# Mesolimbic Dopamine Dysregulation is Linked to Cognitive and Behavioral Deficits in the P301S Model of Tauopathy

**DOI:** 10.1002/alz70855_096712

**Published:** 2025-12-23

**Authors:** Alyson M Curry, Katherine M Holleran, Sara R Jones

**Affiliations:** ^1^ Translational Neuroscience, Winston Salem, NC, USA

## Abstract

**Background:**

Alzheimer's disease (AD) is a progressive neurodegenerative disorder that is characterized by molecular pathologies, the accumulation of amyloid‐β and neurofibrillary tau, and deficits in memory and cognition. Neuropsychiatric symptoms (NPS), such as depression and apathy, are increasingly viewed as early manifestations of AD and have been linked to disease development and progression. Dopamine, a critical regulator of motivation, learning, and memory, has been implicated in AD pathophysiology, but its specific role in disease progression remains underexplored. This study investigates the relationship between cognitive deficits and mesolimbic dopamine function in Tau P301S mice, a model of tauopathy.

**Method:**

Cognitive and behavioral assessments were conducted in male and female Tau P301S mice at 3, 6, and 9 months of age using novel object recognition (NOR), social interaction, and sucrose preference tests. Mesolimbic dopamine function was evaluated through ex vivo fast‐scan cyclic voltammetry in the nucleus accumbens core.

**Result:**

Cognitive deficits were evident as early as 3 months in Tau P301S mice. By 6 months, these mice exhibited impaired NOR performance and reduced sucrose preference. Although dopamine release and reuptake remained unchanged at this stage, there was reduced D2 receptor sensitivity that correlated with cognitive deficits. At 9 months, Tau P301S mice demonstrated worsened behavioral and cognitive impairments, accompanied by trending reductions in dopamine release and reuptake, as well as further declines in D2 receptor sensitivity. Notably, increased NOR performance positively correlated with dopamine release. A small cohort of APP/PS1 mice displayed similar deficits in behavior and dopamine function.

**Conclusion:**

Tau P301S mice exhibit progressive cognitive and social impairments that correlate with alterations in mesolimbic dopamine function. Similar dysfunctions were observed in APP/PS1 mice, underscoring the relevance of dopamine dysregulation across multiple AD models. These findings highlight the role of mesolimbic dopamine system dysfunction in AD and suggest targeting dopamine‐related pathways to alleviate cognitive and behavioral deficits.

